# Cognition Impairment Prior to Errors of Working Memory Based on Event-Related Potential

**DOI:** 10.3389/fnbeh.2019.00013

**Published:** 2019-02-12

**Authors:** Yi Xiao, Jintao Wu, Weicai Tang, Chenhui Sun, Feng Ma, Lingling Guo

**Affiliations:** ^1^National Key Laboratory of Human Factors Engineering, China Astronaut Research and Training Center, Beijing, China; ^2^School of Biological Science and Medical Engineering, Beihang University, Beijing, China; ^3^School of Aerospace Engineering, Tsinghua University, Beijing, China; ^4^Department of Psychology, Zhejiang Sci-Tech University, Hangzhou, China

**Keywords:** attention, cognitive control, cognition impairment, memory updating, N2, P3, working memory

## Abstract

Cognitive impairment contributes to errors in different tasks. Poor attention and poor cognitive control are the two neural mechanisms for performance errors. A few studies have been conducted on the error mechanism of working memory. It is unclear whether the changes in memory updating, attention, and cognitive control can cause errors and, if so, whether they can be probed at the same time in one single task. Therefore, this study analyzed event-related potentials in a two-back working memory task. A total of 40 male participants finished the task. The differences between the error and the correct trials in amplitudes and latencies of N1, P2, N2, and P3 were analyzed. The P2 and P3 amplitudes decreased significantly in the error trials, while the N2 amplitude increased. The results showed that impaired attention, poor memory updating, and impaired cognitive control were consistently associated with the error in working memory. Furthermore, the results suggested that monitoring the neurophysiological characteristics associated with attention and cognitive control was important for studying the error mechanism and error prediction. The results also suggested that the P3 and N2 amplitudes could be used as indexes for error foreshadowing.

## Introduction

Performance errors may have serious consequences. For instance, the error made by Apollo astronauts almost led to the collision of the moon with their spacecraft (Shayler, 2000). Further, a serious mistake made by a driver may lead to casualties and loss of the means of transport. Therefore, it is important to study the errors for improving human-system safety. Many causes account for errors, with some defaults in cognitive function playing an important role. Investigating the error-related neural patterns is important to uncover the error mechanisms, thus helping in improving the safety.

The error-related neural mechanisms were usually through analyzing the event-related potentials (ERPs) (Ridderinkhof et al., 2003; Hajcak et al., 2005; Padilla et al., 2006; Maidhof et al., 2009; Masaki et al., 2012; Wessel et al., 2012; Bode and Stahl, 2014; Ora et al., 2015; Shou et al., 2015). Lapse of attention (Padilla et al., 2006; Hanslmayr et al., 2007; Klimesch et al., 2007; Mathewson et al., 2009; Jensen and Mazaheri, 2010) and decreased cognitive control (Ridderinkhof et al., 2003; Kok et al., 2004; Schmajuk et al., 2006; O’Connell et al., 2009) are the two main error-related neural patterns (Mazaheri et al., 2009; Eichele et al., 2010; Shou et al., 2015). Decreased P3 amplitude in the centroparietal region validating the lapse of sustained attention is the cause of error (Padilla et al., 2006; Hanslmayr et al., 2007; Mathewson et al., 2009; Jensen and Mazaheri, 2010). The diminished N2 amplitude in the error trials showed that the impaired cognitive control was the reason for errors (Kok et al., 2004; Falkenstein, 2006; O’Connell et al., 2009). The N2 amplitude correlated with the cognitive control, reflecting the psychology template mismatch, response inhibition, and selection (Patel and Azzam, 2005; Azizian et al., 2006; Folstein and Van, 2008; Gajewski et al., 2008). Some studies indicated that an increased N2 amplitude also correlated with an impaired cognitive control (Kok, 1990; Pinal et al., 2015). A larger amplitude reflects increased energetic costs (Kok, 1990; Pinal et al., 2015), with more resources allocated and more neurons activated. Generally speaking, more efforts are needed for performing the same task with poor performance, indicating an impairment of the cognitive control.

A few studies explored the error-related neural mechanism (Ora et al., 2015; Shou et al., 2015) by analyzing multiple cognitive processes in one single task. Ora et al. (2015) analyzed the spatiotemporal neural activities during performance errors and found that attentional lapses and inappropriate action impulses caused subsequent performance errors in a d2 task. Shou et al. (2015) studied the error mechanism by the comprehensive analysis of N2 and alpha power in the Stroop task. The results revealed that the alpha power of error increased in the parieto-occipital area under congruent trials, while the N2 amplitude decreased only in incongruent trials. These findings implied that poor sustained attention and poor cognitive control caused errors. As to working memory, what cognitive changes may cause errors is still unknown.

Memory updating is the key process of working memory. The P3 amplitude is associated with memory updating, and its latency is related to match function (Watter et al., 2001; Chen et al., 2008). On the contrary, P3 is closely linked with attention and cognitive resource reallocation. P3a is bound with attention to the selection-driven stimulus, especially the novelty stimulus (Falkenstein et al., 1994; Polich, 2007). In contrast, the P3b (Polich and Heine, 1996; Schapkin and Freude, 2013) is related to the reallocation of cognitive resources and memory. Some studies showed that a few cognitive resources were reallocated to other memory processes, resulting in a longer P3 latency and a decreased P3 amplitude (Polich, 2007; Daffner et al., 2011; Wild-Wall et al., 2011; Saliasi et al., 2013; Gajewski and Falkenstein, 2014) in working memory. One study also showed that latency jitter can decreased P3 amplitude (Aricò et al., 2014). P2 is part of memory information process related to the onset of memory updating (Lenartowicz et al., 2010; Yuan et al., 2016). N2 is regarded as an ERP component related to cognitive control in memory (Daffner et al., 2011; Gajewski and Falkenstein, 2014). N1 is the early component of information process, which remains sensitive to physical features of stimulus and reflects the recognition and code processes (Ritter et al., 1979; Hopf et al., 2002). To date, no studies have reported on the error mechanism of working memory. It is unknown whether memory updating, attention, and cognitive control may change in working memory and cause errors. No studies have identified error-related neural patterns in one single task for working memory. Therefore, the two-back working memory task was adopted in this study to investigate error-related neural patterns and cognition impairment.

A few studies showed the N1 difference in errors. Maybe, the stimulus is identical in correct and error trials. Also, it is too simple for recognition and hardly leads to error. Therefore, N1 was analyzed to verify the hypothesis in the present study. The memory updating is the key cognitive process in working memory. Therefore, the P3 and P2 amplitudes may decrease in error trials. Some studies (Ridderinkhof et al., 2003; Kok et al., 2004; Padilla et al., 2006; Schmajuk et al., 2006; Hanslmayr et al., 2007; Klimesch et al., 2007; Eichele et al., 2008; Mathewson et al., 2009; Mazaheri et al., 2009; O’Connell et al., 2009; Jensen and Mazaheri, 2010; Bode and Stahl, 2014; Ora et al., 2015; Shou et al., 2015) showed that poor attention and poor cognitive control were the error-related neural patterns, leading to changes in P3 and N2. The P2 and P3 amplitudes may decrease in error trials, but the N2 amplitude may increase for impaired cognitive control due to the energy cost theory (Kok, 1990; Pinal et al., 2015).

## Materials and Methods

### Participants

This study was approved by the ethics committee of China Astronaut Research and Training Center. All participants provided written informed consent in accordance with the Declaration of Helsinki. For this study, 40 male participants, aged 19–34 years with an average age of 24 years [standard deviation (SD) = 2.92)] were enrolled. The participants were postgraduate or graduate students with normal or corrected-to-normal vision and without any reported psychiatric disorders. Five participants were excluded because of too many artifacts in electroencephalogram (EEG), and the remaining 31 participants who had more than 20 performance errors were selected for further analysis.

### Two-Back Working Memory Task

**FIGURE 1:**
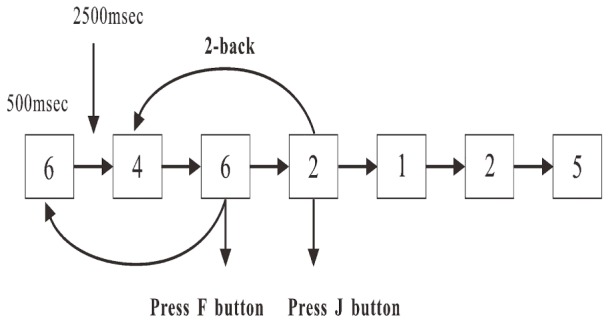
Procedure of two-back working memory task.

A numerical two-back task was used in the present study (Yuan et al., 2016) The sequences of 1, 2, 3, 4, 5, 6, 7, 8, and 9 were pseudo-randomly displayed in a white against black background on a 24-inch LCD screen (with an updating rate of 60 Hz) one by one. The height and width of the stimulus were 1.8 and 1.4 cm, respectively. At the beginning of each trial, a stimulus (Figure 1) was displayed for 500 ms or disappeared if responded. Then, a new stimulus was displayed after 2500 ms. Participants were required to press the “F” or “J” button as accurately and quickly as possible. If the stimulus presented was the same as the one that had appeared two presentations before, the participants were required to press the button “F”; if not, the participants were required to press the button “J.” The time of the task was 10 min, and the target rate was 33.33%. The number of trials was different between participants, probably due to the reaction times. However, the minimum number was 200 from the required time in the task design.

### Procedure

The participants were needed to come to the laboratory two times. The first time was to get training, 1 day before the test, to ensure that all participants were familiar with the task. The second time was to get the formal test the next day. EEG data were recorded during the working memory test. The laboratory had a shield room. The participants were seated in the shield room to record the EEG data. The size of the shield room was 2.4 m × 2.4 m × 2.4 m.

### EEG Recording

Electroencephalogram data were recorded during the working memory test. After placement of electrodes, the participants were requested to sit in a comfortable chair 80 cm away from the LCD screen and instructed to refrain from excessive blinking and movement during data collection. Participants then underwent a two-back working memory task. Then, the EEG was recorded with Ag-AgCl electrodes embedded in an elastic cap (EasyCap, Brain Products GmbH) at 63 scalp locations according to the 10–20 system of Jasper (1958). The vertical electrooculogram (vEOG) was recorded from an electrode infraorbital to the left eye and the horizontal EOG was recorded at the outer canthi of the right eye. The ground electrode was positioned at AFz, and the reference electrode was positioned at FCz. The Vertical and horizontal electrooculogram (EOG) were recorded from electrodes above and below the right eye and on the outer canthi of both eyes. The electrode impedance was kept below 5 kΩ. Signals were recorded using the BrainVision Recorder (Brain Products GmbH, Ver. 1.03), with a band-pass filtered at 0.01–250 Hz. The sampling rate was 1000 Hz, and the signals were amplified in the range of ±3.27 mV, and at a resolution of 0.1 μV.

### Statistical Analysis of Behavioral Data

Descriptive statistics of error rate, reaction times (RTs) of correct and error responses, and the total number of errors were computed. The error was that the response did not accord to the stimulus. For example, although the button accorded to the stimulus was “F”, the participant pressed “J”; this was an error response. The omission was not included in error statistics. The error rate was defined as the number of error responses divided by the total number of responses. RTs of two trials (correct vs. error) were examined using the paired *t*-test (Hsieh et al., 2010; Murphy et al., 2010; Sambrook and Goslin, 2014; Ora et al., 2015; Shou et al., 2015).

### EEG Analysis

The EEG data were processed off-line using the BrainVision Analyzer 2.0. Software (Brain Products GmbH, German). The mastoids (TP9, TP10) were selected as the new references (Brain Products GmbH; Möckel et al., 2015). The semi-automatic inspection method was implemented to inspect the raw data. The gradient criterion was set at 50 μV/ms. The maximum absolute difference allowed was 200 μV with a 200-ms interval. The amplitude was in the range of -200 to 200 μV. The allowed activity with the lowest amplitude was 0.5 μV. After inspecting the raw data, the EEG signals were corrected for eye movement artifacts using the artifact rejection method, which was based on the Gratton and Coles’ algorithm. This procedure was implemented in the BrainVision Analyzer 2.0 Software. The continuous EEG signals were filtered using a band-pass filter from 0.1 to 35 Hz with a 0-phase shift of 48 dB, and the notch filter was 50 Hz.

Stimulus-locked data were segmented into epochs of -200 ms to 800 ms after stimulus and were baseline-corrected relative to the interval of -200 ms to 0 ms. The ERPs of accepted trials were then averaged separately for obtaining a correct or error trial. The N1, P2, N2, and P3 values were quantified as the maximum amplitudes, and the intervals were increased from 130 to 170 ms, 170 to 270 ms, 270 to 360 ms, and 400 to 500 ms post-stimulus, respectively. The maximum amplitudes of each ERP component were the average of 10 points around the peak amplitude. Grand average waveforms of N1, N2, and P3 were created for the correct and error trials at each of the three electrode sites (Fz, Cz, and Pz). The three components were analyzed using a two-way repeated-measures analysis of variance with Trial (correct and error) and Site (Fz, Cz, and Pz) as within-subject factors (Gehring and Fencsik, 2001). With no overt P2 in Pz, ANOVA with repeated measures was conducted on P2 with Trial (correct and error) and Site (Fz and Cz) as within-subject variables. The partial eta squared ($\eta_{p}^{2}$) was the effect size, and the Greenhouse–Geisser correction was used where appropriate (Liu et al., 2015). Paired-sample *t*-tests were used for analysis of latencies of Trial (correct and error).

## Results

### Behavioral Results

**FIGURE 2:**
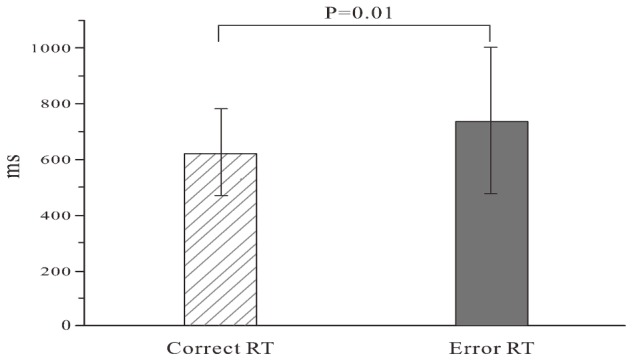
Reaction time of error and correct trials.

Due to different RTs, the response number of the participants was between 200 and 422 within 10 min and the mean response number was 356.4 (SD = 59.8). The mean error rate was 0.12 (SD = 0.05) with the 0.01 (SD = 0.02) omission rate, and the mean error responses in 31 participants was 43.8 (SD = 18.6). The RTs of error trials were significantly longer than those of correct trials (*t* = -2.078; *P* = 0.01). The results are shown in Figure 2.

### ERP Results

**FIGURE 3:**
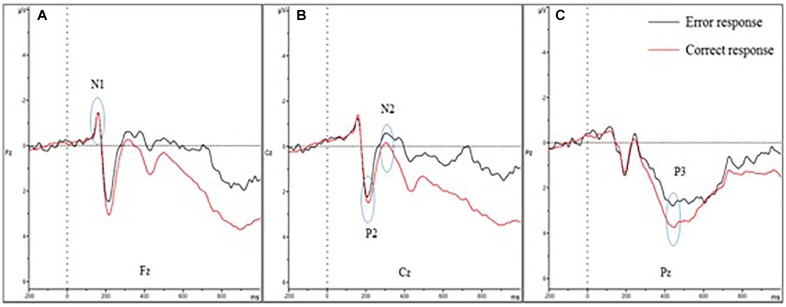
Event-related potential of correct and error trials of all participants. **(A–C)** show the N1, P2, N2, and P3 amplitudes of the two trials. **(A)** Shows the four amplitudes in Fz; **(B)** shows the four amplitudes in Cz; and **(C)** shows the four amplitudes in Pz. The N1 amplitude showed no difference between error and correct trials. The P2 and P3 amplitudes diminished in error trials, while the N2 amplitude was larger.

**FIGURE 4:**
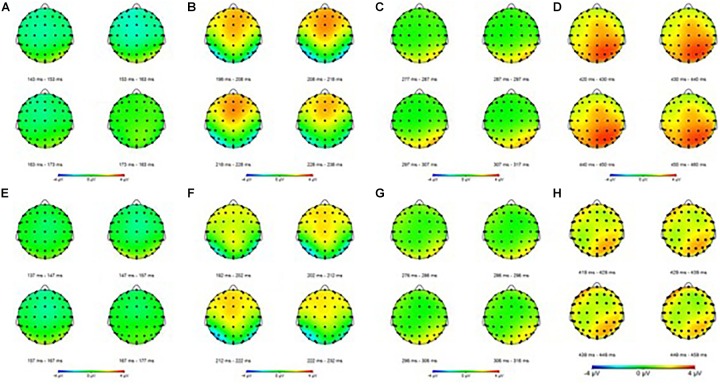
Event-related potential mapping of correct and error trials. **(A–D)** Show the N1, P2, N2, and P3 mapping of correct ERP, respectively; **(E–G)** show the N1, P2, N2, and P3 mapping of error ERP, respectively. **(A,E)** Show the N1 amplitudes of the two trials; **(B,F)** show that the P2 amplitudes of the error trials was smaller than that of the correct trials in central areas; **(C,G)** show the N2 amplitudes of the two trials; **(D,H)** show that the P3 amplitudes of the error trials was smaller than that of the correct trials in the parietal areas.

The means of ERP amplitudes and latencies are shown in Tables 1, 2. The ERP wave and mapping of correct and error trials of all participants are presented in Figure 3, 4.

**Table 1:** Event-related potential amplitudes.

Electrode site		Fz		Cz		Pz	
			
Trial		Correct	Error	Correct	Error	Correct	Error
N1	Mean	-1.82	-1.92	-1.72	-1.81	-0.94	-1.11
	SD	1.20	1.42	1.33	1.40	1.11	1.54
N2	Mean	-1.06	-1.51	-1.13	-1.86	-1.03	-1.32
	SD	2.35	2.85	2.15	2.55	1.72	1.99
P2	Mean	3.84	3.31	3.40	3.06	/	/
	SD	1.76	1.99	1.79	1.78	/	/
P3	Mean	1.57	1.01	2.38	1.48	4.32	3.49
	SD	3.02	3.34	3.12	3.35	2.68	2.09


**Table 2:** Event-related potential latency.

ERP	Correct	Error	*t*	*P*
N1	155.29 ± 12.028	152.68 ± 11.976	-0.86	0.66
N2	294.03 ± 33.355	289.87 ± 31.678	-0.50	0.89
P2	209.45 ± 20.772	210.32 ± 23.798	0.15	0.64
P3	433.13 ± 30.5	429.06 ± 34.112	-0.50	0.52


For the N1 amplitudes, no main effect of trial was observed [*F*(1,30) = 0.386; *P* = 0.539; $\eta_{p}^{2}$ = 0.013], but a main effect of electrode sites was found [*F*(1.207,36.207) = 8.260; *P* = 0.004; $\eta_{p}^{2}$ = 0.216)] and the Fz showed the largest amplitude. In contrast, the Pz amplitude was the smallest. However, no interaction effect was noted between electrode sites and trials [*F*(1.385,41.546) = 0.141; *P* = 0.790; $\eta_{p}^{2}$ = 0.005].

The P2 amplitudes decreased in error trials significantly [*F*(1,30) = 4.211; *P* = 0.049; $\eta_{p}^{2}$ = 0.123]. Moreover, a main effect of electrode sites was observed [*F*(1,30) = 4.506; *P* = 0.042; $\eta_{p}^{2}$ = 0.131]. The Fz amplitude was the largest, but the Cz amplitude was the smallest. However, no significant interaction effect was observed between the electrode sites and the trials [*F*(1,30) = 2.244; *P* = 0.145; $\eta_{p}^{2}$ = 0.070].

For the N2 amplitudes, the amplitude in the error trial increased significantly [*F*(1,30) = 5.142; *P* = 0.031; $\eta_{p}^{2}$ = 0.146]. However, no main effect of electrode sites was found [*F*(1.185,35.551) = 0.290; *P* = 0.632; $\eta_{p}^{2}$ = 0.010]. Additionally, no interaction effect was observed between electrode sites and trials [*F*(1.076,32.392) = 0.533; *P* = 0.483; $\eta_{p}^{2}$ = 0.017].

For the P3 amplitudes, the amplitudes of error trial decreased significantly [*F*(1,30) = 6.663; *P* = 0.015; $\eta_{p}^{2}$ = 0.182]. Furthermore, a main effect of electrode sites was noted [*F*(1.240,37.191) = 10.607; *P* = 0.001; $\eta_{p}^{2}$ = 0.261]. The Pz (P3b) amplitude was the largest, but the Fz (P3a) amplitude was the smallest. However, no significant interaction effects were found between the electrode sites and the trials [*F*(1.335,40.047) = 0.660; *P* = 0.464; $\eta_{p}^{2}$ = 0.022]. The mean amplitudes of all participants (Figure 3) showed that the amplitudes in correct trial were far larger than the errors, especially in the central and parietal areas. These results were also illustrated using topographic maps (Figure 4).

The *t*-test of latency in different trials (correct vs. error) for N1, N2, P2, and P3 revealed no significant difference (N1: *t* = 1.40, *P* = 0.17; N2: *t* = 0.82, *P* = 0.42; P2: *t* = -0.24, *P* = 0.81; P3: *t* = -0.67, *P* = 0.51).

## Discussion

The present study partially verified the hypotheses. Reduced P2 and P3 amplitudes in error trials were found. However, the N2 amplitudes increased in error trials. The RT of the error trial was longer than that of the correct trial. The results revealed that poor attention, poor cognitive control, and impaired memory updating might cause errors in working memory. The N1 amplitudes showed no difference between the two trials.

As expected, the N1 amplitudes showed no significant changes in different trials. N1 is thought to be the early component of information processing. Its amplitudes reflect the recognition and code processing of stimulus (Kutas and Hillyard, 1980; Hopf et al., 2002), which are sensitive to the physical features of the stimulus. Therefore, the results indicated that the neural activity was comparable during the stimulus encoding stage of working memory process between correct and error trials. Additionally, no differences were found between trials that resulted from the same too simple physical features of the stimulus.

No study has been found regarding the association of P2 with an error. The results revealed that the P2 amplitude decreased when participants presented an error response. Previous studies found that the P2 component reflected the initial stage of context updating, and was believed to be the onset of memory updating (Lenartowicz et al., 2010; Yuan et al., 2016). Therefore, the diminished amplitudes of error trials were related to the impaired ability of memory updating onset. The onset of updating was encoded after stimuli and then translated to phonological representations. Thus, it was considered one of the crucial steps for successful performance and impaired ability, resulting in an error response in this study.

The increased N2 amplitude in error trial implied that the cognitive control decreased (Kok, 1990; Caseras et al., 2006; Unsworth et al., 2009; Bode and Stahl, 2014; Pinal et al., 2015) and induced errors based on the energy cost theory (Kok, 1990; Pinal et al., 2015). N2 was related to cognitive control, including response selection and conflict detection in memory (Daffner et al., 2011; Gajewski and Falkenstein, 2014). The peak N2 amplitude was significantly larger in the older group than in the younger group (Pinal et al., 2015), which might be due to the use of more energy for the older participants. The increased N2 amplitude reflected an enhanced energetic cost in cognitive control, such as mismatch and response selection (Nieuwenhuis et al., 2005; Folstein and Van, 2008; Daffner et al., 2011; O’connell et al., 2012b). Previous studies showed that a high N2 amplitude was found in patients than in controls because the patients need more resources to process the information including the mismatch and response selection (Bruder et al., 1998, 2001; Daurignac et al., 2006; Guillem et al., 2006; Shu et al., 2014; Sumich et al., 2014; Pinal et al., 2015; Zuj et al., 2017). A larger N2 amplitude possibly demanded greater efforts in task completion and even induced hyper-activation by way of more efforts during the completion of a relatively simple task (Bruder et al., 1998, 2001; Guillem et al., 2006; Sumich et al., 2014). Some studies showed a diminished P3 amplitude in working memory because more resources were recruited in the cognitive control and increased the N2 amplitude (Daffner et al., 2011; Gajewski and Falkenstein, 2014). More neuron-related cognitive control was activated, subsequently increasing the N2 amplitude. The stimulus was identical in all the error and correct trials, and therefore the cognitive demands remained the same. This indicated that more resources were used for the same task, and the error rate increased, indicating a declination in cognitive ability in the wrong trial.

P3 was associated with sustained attention, cognitive resource reallocation (Snyder and Hillyard, 1976; Donchin, 1981; Nieuwenhuis et al., 2005; Polich and Criado, 2006; Polich, 2007; Verleger, 2008; Duncan et al., 2009; Beste et al., 2010; Hart et al., 2012; O’Connell et al., 2012a; Kim and Kim, 2016), and memory updating (Donchin and Coles, 1988; Polich, 2007; Zhao et al., 2013).

### Decreased P3 Amplitude Reflected Impaired Attention

Falkenstein and Polich (Falkenstein et al., 1994; Polich, 2007) showed that frontal P3(P3a) was related to attention selection (Polich and Heine, 1996; Schapkin and Freude, 2013) and parietal P3(P3b) was associated with cognitive resource reallocation. The decreased P3 amplitude in frontal and parietal areas implied that some resources were reallocated in other memory processes, impairing the attention. Gajewski and Falkenstein (2014) found that cognitive resource was reallocated in other processes and the impaired attention led to a decreased P3 amplitude in the two-back working memory task (Polich and Heine, 1996; Polich, 2007; Daffner et al., 2011; Wild-Wall et al., 2011; Saliasi et al., 2013; Schapkin and Freude, 2013). A decline of P3 amplitude in frontal and parietal areas implied impaired attention and diminished cognitive resources, resulting in increased omission rate, false alarm, and more errors (Donchin and Coles, 1988; Polich, 2007; Zhao et al., 2013; Schapkin and Freude, 2014). On the contrary, longer RT in error trials was thought as attention lapses, which was supported by fMRI (Weissman et al., 2006) and other findings (Padilla et al., 2006). This was consistent with the results of the present study, which implied impaired attention.

### Decreased P3 Amplitude Reflected Deficit Ability in Memory Updating

The P3 amplitude is an effective measurement of memory updating (Donchin and Coles, 1988; Watter et al., 2001; Polich, 2007; Chen et al., 2008; Zhao et al., 2013), and the latency is related to the match function (Donchin and Coles, 1988; Watter et al., 2001; Polich, 2007; Chen et al., 2008; Zhao et al., 2013). The ability associated with memory updating increased with better performance, leading to an increased P3 amplitude in the parietal area when the participants underwent n-back training (Watter et al., 2001; Chen et al., 2008). A reduced amplitude indicated that the memory updating capability was impaired. Updating included code, operation, search, and selection of information. Impaired memory updating caused some faults in information operation and updating in the memory mode. Thus, the errors were related with poor memory updating.

## Conclusion

Diminished P3 and P2 amplitudes and an increased N2 amplitude in errors were related to impaired attention and deficit in memory updating and cognitive control. The diminished P3 amplitude indicated that more cognitive resources were reallocated to other memory processes, leading to impaired attention in the memory updating process. As a result, it caused a deficit in the ability of memory updating and decreased cognitive control. This was the first study on the poor memory updating causing errors.

Poor attention and cognitive control were the major causes of error in many common tasks (Ridderinkhof et al., 2003; Kok et al., 2004; Padilla et al., 2006; Schmajuk et al., 2006; Hanslmayr et al., 2007; Klimesch et al., 2007; Mathewson et al., 2009; O’Connell et al., 2009; Jensen and Mazaheri, 2010). Hence, much attention should be paid to the neurocognitive characteristics, which are important for error mechanism and can be used as indexes for error prediction. How to predict the error based on EEG trial-to-trial analysis needs further investigation in the future.

## Author Contributions

All authors listed have made a substantial, direct and intellectual contribution to the work, and approved it for publication.

## Conflict of Interest Statement

The authors declare that the research was conducted in the absence of any commercial or financial relationships that could be construed as a potential conflict of interest.

## References

[B1] Aricò P. Aloise F. Schettini F. Salinari S. Mattia D. Cincotti F. (2014). Influence of p300 latency jitter on event related potential-based brain–computer interface performance. *J. Neural Eng.* 11:035008. 10.1088/1741-2560/11/3/035008 24835331

[B2] Azizian A. Freitas A. L. Parvaz M. A. Squires N. K. (2006). Beware misleading cues: perceptual similarity modulates the N2/P3 complex. *Psychophysiology* 43 253–260. 10.1111/j.1469-8986.2006.00409.x 16805863

[B3] Beste C. Willemssen R. Saft C. Falkenstein M. (2010). Response inhibition subprocesses and dopaminergic pathways: basal ganglia disease effects. *Neuropsychologia* 48 366–373. 10.1016/j.neuropsychologia.2009.09.023 19782093

[B4] Bode S. Stahl J. (2014). Predicting errors from patterns of event-related potentials preceding an overt response. *Biol. Psychol.* 103 357–369. 10.1016/j.biopsycho.2014.10.002 25450163

[B5] Bruder G. E. Kayser J. Tenke C. E. Friedman M. Malaspina D. Gorman J. M. (2001). Event-related potentials in schizophrenia during tonal and phonetic oddball tasks: relations to diagnostic subtype, symptom features and verbal memory. *Biol. Psychiatry* 50 447–452. 10.1016/S0006-3223(01)01168-4 11566162

[B6] Bruder G. E. Tenke C. E. Towey J. P. Leite P. Fong R. Stewart J. E. (1998). Brain ERPs of depressed patients to complex tones in an oddball task: relation of reduced P3 asymmetry to physical anhedonia. *Psychophysiology* 35 54–63. 10.1111/1469-8986.3510054 9499706

[B7] Caseras X. Mataix-Cols D. Giampietro V. Rimes K. A. Brammer M. Zelaya F. (2006). Probing the working memory system in chronic fatigue syndrome: a functional magnetic resonance imaging study using the n-back task. *Psychosom. Med.* 68 947–955. 10.1097/01.psy.0000242770.50979.5f 17079703

[B8] Chen Y.-N. Mitra S. Schlaghecken F. (2008). Sub-processes of working memory in the N-back task: an investigation using ERPs. *Clin. Neurophysiol.* 119 1546–1559. 10.1016/j.clinph.2008.03.003 18448388

[B9] Daffner K. R. Chong H. Sun X. Tarbi E. C. Riis J. L. McGinnis S. M. (2011). Mechanisms underlying age-and performance-related differences in working memory. *J. Cogn. Neurosci.* 23 1298–1314. 10.1162/jocn.2010.21540 20617886 PMC3076134

[B10] Daurignac E. Houdé O. Jouvent R. (2006). Negative priming in a numerical Piaget-like task as evidenced by ERP. *J. Cogn. Neurosci.* 18 730–736. 10.1162/jocn.2006.18.5.730 16768373

[B11] Donchin E. (1981). Surprise!… surprise? *Psychophysiology* 18 493–513. 10.1111/j.1469-8986.1981.tb01815.x 7280146

[B12] Donchin E. Coles M. G. (1988). Is the P300 component a manifestation of context updating? *Behav. Brain Sci.* 11 357–374. 10.1016/j.clinph.2009.07.045 19796989

[B13] Duncan C. C. Barry R. J. Connolly J. F. Fischer C. Michie P. T. Näätänen R. (2009). Event-related potentials in clinical research: guidelines for eliciting, recording, and quantifying mismatch negativity. P300, and N400. *Clin. Neurophysiol.* 120 1883–1908. 10.1016/j.clinph.2009.07.045 19796989

[B14] Eichele H. Juvodden H. T. Ullsperger M. Eichele T. (2010). Mal-adaptation of event-related EEG responses preceding performance errors. *Front. Hum. Neurosci.* 4:65. 10.3389/fnhum.2010.00065 20740080 PMC2927308

[B15] Eichele T. Debener S. Calhoun V. D. Specht K. Engel A. K. Hugdahl K. (2008). Prediction of human errors by maladaptive changes in event-related brain networks. *Proc. Natl. Acad. Sci. U.S.A.* 105 6173–6178. 10.1016/j.neuropsychologia.2006.12.014 18427123 PMC2329680

[B16] Falkenstein M. (2006). Inhibition, conflict and the Nogo-N2. *Clin. Neurophysiol. Off. J. Int. Fed. Clin. Neurophysiol.* 117 1638–1640. 10.1016/j.clinph.2006.05.002 16798078

[B17] Falkenstein M. Hohnsbein J. Hoormann J. (1994). Effects of choice complexity on different subcomponents of the late positive complex of the event-related potential. *Electroencephalogr. Clin. Neurophysiol.* 92 148–160. 7511512 10.1016/0168-5597(94)90055-8

[B18] Folstein J. R. Van P. C. (2008). Influence of cognitive control and mismatch on the N2 component of the ERP: a review. *Psychophysiology* 45 152–170. 10.1027/0269-8803/a000123 17850238 PMC2365910

[B19] Gajewski P. D. Falkenstein M. (2014). Age-related effects on ERP and oscillatory EEG-dynamics in a 2-back task. *J. Psychophysiol.* 28 162–177. 10.1016/j.brainres.2007.10.076 18053974

[B20] Gajewski P. D. Stoerig P. Falkenstein M. (2008). ERP–correlates of response selection in a response conflict paradigm. *Brain Res.* 1189 127–134. 10.1016/j.pnpbp.2006.02.009 18053974

[B21] Gehring W. J. Fencsik D. E. (2001). Functions of the medial frontal cortex in the processing of conflict and errors. *J. Neurosci.* 21 9430–9437. 10.1523/JNEUROSCI.21-23-09430.2001 11717376 PMC6763895

[B22] Guillem F. Chouinard S. Poulin J. Godbout R. Lalonde P. Melun P. (2006). Are cholinergic enhancers beneficial for memory in schizophrenia? An event-related potentials (ERPs) study of rivastigmine add-on therapy in a crossover trial. *Prog. Neuro Psychopharmacol. Biol. Psychiatry* 30 934–945. 16580765 10.1016/j.pnpbp.2006.02.009

[B23] Hajcak G. Nieuwenhuis S. Ridderinkhof K. R. Simons R. F. (2005). Error-preceding brain activity: robustness, temporal dynamics, and boundary trails. *Biol. Psychol.* 70 67–78. 10.1016/j.biopsycho.2004.12.001 16168251

[B24] Hanslmayr S. Aslan A. Staudigl T. Klimesch W. Herrmann C. S. Bäuml K. H. (2007). Prestimulus oscillations predict visual perception performance between and within subjects. *Neuroimage* 37 1465–1473. 10.1016/j.neuroimage.2007.07.011 17706433

[B25] Hart E. Dumas E. Reijntjes R. van der Hiele K. van den Bogaard S. Middelkoop H. (2012). Deficient sustained attention to response task and P300 characteristics in early Huntington’s disease. *J. Neurol.* 259 1191–1198. 10.1007/s00415-011-6334-0 22143614 PMC3366183

[B26] Hopf J.-M. Vogel E. Woodman G. Heinze H.-J. Luck S. J. (2002). Localizing visual discrimination processes in time and space. *J. Neurophysiol.* 88 2088–2095. 10.1152/jn.2002.88.4.2088 12364530

[B27] Hsieh S. Tsai C. Y. Tsai L. L. (2010). Error correction maintains post-error adjustments after one night of total sleep deprivation. *J. Sleep Res.* 18 159–166. 10.1111/j.1365-2869.2008.00730.x 19645961

[B28] Jasper H. H. (1958). The ten-twenty electrode system of the International Federation. *Electroencephalogr. Clin. Neurophysiol.* 10 370–375. 10.1097/00006534-195205000-00008 10590970

[B29] Jensen O. Mazaheri A. (2010). Shaping functional architecture by oscillatory alpha activity: gating by inhibition. *Front. Hum. Neurosci.* 4:186. 10.3389/fnhum.2010.00186 21119777 PMC2990626

[B30] Kim S. Kim M.-S. (2016). Deficits in verbal working memory among college students with attention-deficit/hyperactivity disorder traits: an event-related potential study. *Clin. Psychopharmacol. Neurosci.* 14 64–73. 10.9758/cpn.2016.14.1.64 26792042 PMC4730935

[B31] Klimesch W. Sauseng P. Hanslmayr S. (2007). EEG alpha oscillations: the inhibition-timing hypothesis. *Brain Res. Rev.* 53 63–88. 10.1016/j.brainresrev.2006.06.003 16887192

[B32] Kok A. (1990). Internal and external control: a two-factor model of amplitude change of event-related potentials. *Acta Psychol.* 74 203–236. 10.1016/0001-6918(90)90006-2 2251929

[B33] Kok A. Ramautar J. R. De Ruiter M. B. Band G. P. Ridderinkhof K. R. (2004). ERP components associated with successful and unsuccessful stopping in a stop-signal task. *Psychophysiology* 41 9–20. 10.1046/j.1469-8986.2003.00127.x 14692996

[B34] Kutas M. Hillyard S. A. (1980). Event-related brain potentials to semantically inappropriate and surprisingly large words. *Biol. Psychol.* 11 99–116. 10.1016/0301-0511(80)90046-0 7272388

[B35] Lenartowicz A. Escobedoquiroz R. Cohen J. D. (2010). Updating of context in working memory: an event-related potential study. *Cogn. Affect. Behav. Neurosci.* 10 298–315. 10.3758/s13415-015-0335-x 20498352 PMC2906710

[B36] Liu Q. Zhou R. Liu L. Zhao X. (2015). Effects of 72hours total sleep deprivation on male astronauts’ executive functions and emotion. *Compr. Psychiatry* 61 28–35. 10.1016/j.comppsych.2015.05.015 26112064

[B37] Maidhof C. Rieger M. Prinz W. Koelsch S. (2009). Nobody is perfect: ERP effects prior to performance errors in musicians indicate fast monitoring processes. *PLoS One* 4:e5032. 10.1371/journal.pone.0005032 19337379 PMC2660409

[B38] Masaki H. Murphy T. I. Kamijo K. Yamazaki K. Sommer W. (2012). Foreshadowing of performance accuracy by event-related potentials: evidence from a minimal-conflict task. *PLoS One* 7:e38006. 10.1371/journal.pone.0038006 22701541 PMC3365114

[B39] Mathewson K. E. Gratton G. Fabiani M. Beck D. M. Ro T. (2009). To see or not to see: pre-stimulus alpha phase predicts visual awareness. *J. Neurosci. Off. J. Soc. Neurosci.* 29 2725–2732. 10.1523/JNEUROSCI.3963-08.2009 PMC2724892 19261866

[B40] Mazaheri A. Nieuwenhuis I. L. van Dijk H. Jensen O. (2009). Prestimulus alpha and mu activity predicts failure to inhibit motor responses. *Hum. Brain Mapp.* 30 1791–1800. 10.1002/hbm.20763 19308934 PMC6870709

[B41] Möckel T. Beste C. Wascher E. (2015). The effects of time on task in response selection – an ERP study of mental fatigue. *Sci. Rep.* 5:10113. 10.1038/srep10113 26054837 PMC4460573

[B42] Murphy T. I. Richard M. Masaki H. Segalowitz S. J. (2010). The effect of sleepiness on performance monitoring: I know what I am doing, but do I care? *J. Sleep Res.* 15 15–21. 10.1111/j.1365-2869.2006.00503.x 16489998

[B43] Nieuwenhuis S. Aston-Jones G. Cohen J. D. (2005). Decision making, the P3, and the locus coeruleus–norepinephrine system. *Psychol. Bull.* 131 510–532. 10.1037/0033-2909.131.4.510 16060800

[B44] O’Connell R. G. Balsters J. H. Kilcullen S. M. Campbell W. Bokde A. W. Lai R. (2012a). A simultaneous ERP/fMRI investigation of the P300 aging effect. *Neurobiol. Aging* 33 2448–2461. 10.1016/j.neurobiolaging.2011.12.021 22277263

[B45] O’Connell R. G. Dockree P. M. Bellgrove M. Turin A. Ward S. Foxe J. (2009). Two types of action error: electrophysiological evidence for separable inhibitory and sustained attention neural mechanisms producing error on go/no-go tasks. *J. Cogn. Neurosci.* 21 93–104. 10.1162/jocn.2009.21008 18476764

[B46] O’connell R. G. Dockree P. M. Kelly S. P. (2012b). A supramodal accumulation-to-bound signal that determines perceptual decisions in humans. *Nat. Neurosci.* 15 1729–1735. 10.1038/nn.3248 23103963

[B47] Ora H. Sekiguchi T. Miyake Y. (2015). Dynamic scalp topography reveals neural signs just before performance errors. *Sci. Rep.* 5:12503. 10.1038/srep12503 26289925 PMC4542339

[B48] Padilla M. Wood R. Hale L. Knight R. (2006). Lapses in a prefrontal-extrastriate preparatory attention network predict mistakes. *J. Cogn. Neurosci.* 18 1477–1487. 10.1162/jocn.2006.18.9.1477 16989549

[B49] Patel S. H. Azzam P. N. (2005). Characterization of N200 and P300: selected studies of the event-related potential. *Int. J. Med. Sci.* 2 147–154. 10.7150/ijms.2.147 16239953 PMC1252727

[B50] Pinal D. Zurrón M. Díaz F. (2015). Age-related changes in brain activity are specific for high order cognitive processes during successful encoding of information in working memory. *Front. Aging Neurosci.* 7:75. 10.3389/fnagi.2015.00075 26029099 PMC4426757

[B51] Polich J. (2007). Updating P300: an integrative theory of P3a and P3b. *Clin. Neurophysiol.* 118 2128–2148. 10.1016/j.clinph.2007.04.019 17573239 PMC2715154

[B52] Polich J. Criado J. R. (2006). Neuropsychology and neuropharmacology of P3a and P3b. *Int. J. Psychophysiol.* 60 172–185. 10.1016/j.ijpsycho.2005.12.012 16510201

[B53] Polich J. Heine M. R. (1996). P300 topography and modality effects from a single-stimulus paradigm. *Psychophysiology* 33 747–752. 10.1111/j.1469-8986.1996.tb02371.x 8961797

[B54] Ridderinkhof K. R. Nieuwenhuis S. Bashore T. R. (2003). Errors are foreshadowed in brain potentials associated with action monitoring in cingulate cortex in humans. *Neurosci. Lett.* 348 1–4. 10.1016/S0304-3940(03)00566-4 12893411

[B55] Ritter W. Simson R. Vaughan H. G. Friedman D. (1979). A brain event related to the making of a sensory discrimination. *Science* 203 1358–1361. 10.1126/science.424760 424760

[B56] Saliasi E. Geerligs L. Lorist M. M. Maurits N. M. (2013). The relationship between P3 amplitude and working memory performance differs in young and older adults. *PLoS One* 8:e63701. 10.1371/journal.pone.0063701 23667658 PMC3646823

[B57] Sambrook T. D. Goslin J. (2014). Mediofrontal event-related potentials in response to positive, negative and unsigned prediction errors. *Neuropsychologia* 61 1–10. 10.1016/j.neuropsychologia.2014.06.004 24946315

[B58] Schapkin S. A. Freude G. (2013). Cardiovascular costs of working memory performance: effects of age and performance feedback. *Ind. Health* 51 386–397. 10.2486/indhealth.2012-0203 23518605

[B59] Schapkin S. A. Freude G. (2014). “Neuronal mechanisms of working memory performance in younger and older employees,” in *Proceedings of the International Conference on Engineering Psychology and Cognitive Ergonomics*, (Berlin: Springer), 70–81. 10.1007/978-3-319-07515-0_8

[B60] Schmajuk M. Liotti M. Busse L. Woldorff M. G. (2006). Electrophysiological activity underlying inhibitory control processes in normal adults. *Neuropsychologia* 44 384–395. 10.1016/j.neuropsychologia.2005.06.005 16095637

[B61] Shayler D. (2000). *Disasters and Accidents in Manned Spaceflight.* Berlin: Springer.

[B62] Shou G. Dasari D. Ding L. (2015). Pre-stimulus alpha and post-stimulus N2 foreshadow imminent errors in a single task. *Neuropsychologia* 77 346–358. 10.1016/j.neuropsychologia.2015.09.006 26362494

[B63] Shu I.-W. Onton J. A. Prabhakar N. O’Connell R. M. Simmons A. N. Matthews S. C. (2014). Combat veterans with PTSD after mild TBI exhibit greater ERPs from posterior–medial cortical areas while appraising facial features. *J. Affect. Disord.* 155 234–240. 10.1016/j.jad.2013.06.057 24342149

[B64] Snyder E. Hillyard S. A. (1976). Long-latency evoked potentials to irrelevant, deviant stimuli. *Behav. Biol.* 16 319–331. 10.1016/S0091-6773(76)91447-4 1275853

[B65] Sumich A. Castro A. Kumari V. (2014). N100 and N200, but not P300, amplitudes predict paranoia/suspiciousness in the general population. *Pers. Individ. Differ.* 61 74–79. 10.1016/j.paid.2014.06301.009

[B66] Unsworth N. Redick T. S. Heitz R. P. Broadway J. M. Engle R. W. (2009). Complex working memory span tasks and higher-order cognition: a latent-variable analysis of the relationship between processing and storage. *Memory* 17 635–654. 10.1080/09658210902998047 19536691

[B67] Verleger R. (2008). P3b: towards some decision about memory. *Clin. Neurophysiol.* 119 968–970. 10.1016/j.clinph.2007.11.175 18222107

[B68] Watter S. Geffen G. M. Geffen L. B. (2001). The n-back as a dual-task: P300 morphology under divided attention. *Psychophysiology* 38 998–1003. 10.1111/1469-8986.3860998 12240676

[B69] Weissman D. H. Roberts K. C. Visscher K. M. Woldorff M. G. (2006). The neural bases of momentary lapses in attention. *Nat. Neurosci.* 9 971–978. 10.1038/nn1727 16767087

[B70] Wessel J. R. Danielmeier C. Morton J. B. Ullsperger M. (2012). Surprise and error: common neuronal architecture for the processing of errors and novelty. *J. Neurosci. Off. J. Soc. Neurosci.* 32 7528–7537. 10.1523/JNEUROSCI.6352-11.2012 22649231 PMC6703591

[B71] Wild-Wall N. Falkenstein M. Gajewski P. D. (2011). Age-related differences in working memory performance in A 2-back task. *Front. Psychol.* 2:186. 10.3389/fpsyg.2011.00186 21909328 PMC3163893

[B72] Yuan Y. Leung A. W. Duan H. Zhang L. Zhang K. Wu J. (2016). The effects of long-term stress on neural dynamics of working memory processing: an investigation using ERP. *Sci. Rep.* 6:23217. 10.1038/srep23217 27000528 PMC4802387

[B73] Zhao X. Zhou R. Fu L. (2013). Working memory updating function training influenced brain activity. *PLoS One* 8:e71063. 10.1371/journal.pone.0071063 24015182 PMC3754993

[B74] Zuj D. V. Felmingham K. L. Palmer M. A. Lawrence-Wood E. Van Hooff M. Lawrence A. J. (2017). Neural activity and emotional processing following military deployment: effects of mild traumatic brain injury and posttraumatic stress disorder. *Brain Cogn.* 118 19–26. 10.1016/j.bandc.2017.07.001 28738210

